# Recent advances in understanding spondyloarthritis

**DOI:** 10.12688/f1000research.10739.1

**Published:** 2017-03-22

**Authors:** J.S.H. Gaston

**Affiliations:** 1 Department of Medicine, Addenbrooke’s Hospital, Cambridge, UK

**Keywords:** spondyloarthritis, enthesitis, HLA-B27, ERAP1, gut microbiome

## Abstract

This review is concerned with a number of recent publications that contribute to current thinking on the pathogenesis of spondyloarthritis. The areas covered include the lymphocyte population in the enthesis, which is thought to drive enthesitis, and hence clinical manifestations. The debate on how HLA-B27 is implicated in inflammation is also considered, together with recent and contradictory evidence on the effects of the peptide-trimming enzyme ERAP1 on B27 expression and hence susceptibility to spondylitis. Lastly, a recent report on the role of the gut microbiome in an important model of spondyloarthritis is considered.

## Introduction

A great deal of evidence, particularly from genome-wide association studies, has implicated the IL-23–IL-17 cytokine axis in the pathogenesis of spondyloarthritis (SpA). These indications have been strengthened by the success of therapies targeting these cytokines in various forms of SpA: for example, IL-17 blockade in ankylosing spondylitis (AS) and anti-IL-12/-23 in psoriatic arthritis. However, in this context, a number of important questions arise, and I will deal with three:

1. What kind of IL-23-responsive and IL-17-producing cell drives inflammation in SpA, and where is the cell located?

2. How is HLA-B27 implicated in causing inflammation, and how does it affect the IL-23–IL-17 axis?

3. What is the stimulus for the excessive production of IL-23 that drives the IL-23-responsive pro-inflammatory cell?

A number of recent papers have shed light on these questions or raised new points to consider.

## The effector cell in SpA pathogenesis

### Entheseal T cell populations

When Sherlock, Cua, and colleagues ^1^ over-expressed IL-23 in mice (a specific strain, B10.RIII), they noted that this resulted in enthesitis. They also discovered that the normal enthesis contains a T cell population that expresses the IL-23 receptor (IL-23R) and that these cells expand under the influence of excess IL-23. The cells comprised an unusual CD3 ^+^ T cell subset expressing neither CD4 nor CD8. This paper was a considerable advance in our understanding of SpA pathogenesis and has justifiably received a great deal of attention and expectation of confirmative and follow-up studies. One such study has now been published and has used broadly similar methods to those of Sherlock *et al.*: labelling of specific cell populations by coupling their expression of molecules of interest (e.g. IL-23R) to a fluorescent protein (“reporter mice”) so that the cells can be visualised in living tissue by two-photon microscopy ^2^. When the authors labelled all cells expressing the δ chain of the γδ T cell receptor (TCR) in B10.RIII mice, they noted a positive population in entheses (Achilles tendon insertion and vertebral bodies). The cells appeared to be non-motile, rather like the γδ T cell population that is seen in murine skin, where it is believed to perform an immunosurveillance function. This population accounted for approximately 25% of the entheseal T cell population; thus, whilst TCR αβ cells were also clearly present in entheses, the proportion of γδ cells was very high as compared with other sites such as the spleen, where γδ cells represent only 1–2% of T cells. Nevertheless, the normal enthesis contained only a few hundred of these cells. Importantly, the cells mainly expressed Vγ6 as part of the γδ TCR, and such cells have been previously noted to be IL-17 producing. Other characteristics also pointed to this capacity, such as the expression of CCR6 (the cells described by Sherlock *et al.* were CCR6 negative). Additional experiments aimed to characterise the entheseal population further by labelling cells expressing the transcription factor required for IL-17 production, RORγt, or, as in the Sherlock study, IL-23R. This showed that, whilst cells expressing either RORγt or IL-23R were less numerous than those expressing TCR γδ (50% and 25% of the numbers, respectively), the γδ cells still accounted for the majority of IL-23R ^+^ and IL-17-producing cells. Finally, over-expressing IL-23 using the same technique as Sherlock *et al.* resulted in enthesitis and an expansion of the γδ population, and the same expansion was seen at other enthesis-like sites in the body known to be involved in SpA: the aortic root and the ciliary body of the eye.

How do these findings compare with those previously reported? There is agreement that the enthesis in mice contains CD4 ^–^CD8 ^–^ T cells and that there are both TCRαβ ^+^ and TCRγδ ^+^ cells. The new report emphasises the role of a γδ ^+^ subset in relation to IL-23R expression (and hence an ability to respond to raised IL-23 levels) and therefore IL-17 production. There is currently no evidence to determine whether TCRγδ or TCRαβ cells are principally responsible for enthesitis or indeed whether both subsets contribute. Differences in mouse colonies and their gut flora might conceivably affect which T cell subset predominates.

The broader question concerns the normal physiologic role of entheseal T cells and whether such a population is also found in humans. Answers to the latter question are eagerly awaited, but the technical difficulties are formidable. There are major differences between mice and humans in the T cell populations that predominate at different anatomical sites – the so-called “resident lymphocyte populations”. For instance, murine skin mainly contains a particular subset of TCRγδ cells (Vγ3Vδ1 cells), whereas in humans this is absent. Likewise, in the liver, mice have a predominant resident population of invariant natural killer (NK) T (iNKT) cells that recognise bacterial lipids presented by the non-classical MHC-like molecule CD1d, whereas human livers have many fewer iNKT cells and instead a large population of cells – mucosal-associated invariant T cells (MAIT cells) – that recognise certain bacterial metabolites presented by another MHC class I-like molecule, MR1 ^3^. Whilst in skin and portal tracts the resident lymphocyte populations in both species probably detect bacterial products as part of their barrier function, this seems unlikely to be the case in the enthesis, which would not normally be exposed to bacteria. However, even in barrier sites, these resident populations can also recognise alternative ligands generated by local tissue damage rather than by pathogens. In the enthesis, detection of tissue damage would be appropriate, given its function and its exposure to mechanical stress. It will be interesting to determine whether the resident lymphocyte population in the normal human enthesis has a restricted set of receptors geared to the recognition of a product of e.g. mechanical stress. The nature of the “antigen” or metabolite produced in response to stress and recognised by the entheseal population will also be important to determine. However, it is also a feature of resident T cell populations that they can be activated by cytokines as well as by the entity recognised by their invariant receptors; IL-23 has a particular role in this respect.

### Factors altering T cell differentiation in SpA

Whilst a cell expressing the transcription factor RORγt is clearly implicated in the work on entheseal populations, the role in AS of another transcription factor that determines T cell function, Tbet ( *TBX21*), has been less clear. A single nucleotide polymorphism (SNP) in the *TBX21* gene is associated with increased risk of AS, and Lau *et al.* ^4^ recently showed that AS patients homozygous for this SNP had higher levels of Tbet mRNA, though surprisingly this was not the case in healthy controls of the same genotype. Whilst this might suggest that inflammation is needed for the effects of the SNP on expression levels to be manifest, levels in AS patients were independent of disease activity. Proportions of Tbet ^+^ cells were also elevated in AS, especially CD8 ^+^ cells, and levels of expression of Tbet were also higher in these cells. These findings are interesting, since it has been postulated, from work in a number of models of autoimmunity, that IL-17-producing cells are not themselves the pathogenic population in such diseases but instead that the critical cells are derived from the Th17 subset and express interferon-γ (IFNγ) as well as, or instead of, IL-17 (“ex-Th17 cells”). AS patients were shown to have increased proportions of cells making both IFNγ and IL-17. This differentiation of Th17 to IFNγ-producing cells might require Tbet, thus explaining the role of the polymorphism in susceptibility to AS. In keeping with this idea, knockout of the Tbet gene in mice alleviated the SpA-like arthritis seen in SKG mice challenged with fungal antigens (in the form of curdlan). However, precisely how Tbet expression favours the emergence of a potentially pathogenic CD8 ^+^ T cell population producing both IL-17 and IFNγ is not yet clear. Whilst Lau *et al.* cited evidence of Tbet inducing IL-23R expression, more recent evidence suggests that RORγt is the principal transcription factor required for this and that Tbet in fact can inhibit IL-23R expression ^5^. Since IL-23 is central to the SpA disease in SKG mice and is also implicated in human SpA, with both genetic and therapeutic evidence, this paradoxical effect of Tbet on IL-23R expression is puzzling and requires further work. Likewise, the expression of Tbet by the entheseal populations described above would be worth investigating.

## How does HLA-B27 influence susceptibility to SpA?

### How do ERAP1 polymorphisms interact with B27?

Three main ideas have dominated thinking on how HLA-B27 exerts its profound effect on susceptibility to SpA. The first, and oldest, is the straightforward one of B27 presenting an “arthritogenic” (or perhaps “enthesitogenic”) peptide to CD8 ^+^ T cells. The other two look to unconventional properties of B27. One is the tendency of the B27 heavy chain to misfold during synthesis in the endoplasmic reticulum (ER) and hence provoke an ER stress response, which in turn influences cytokine secretion, particularly IL-23 ^6^. The other is its ability to be expressed on the cell surface as dimers/multimers that engage receptors (KIR) on T cells, NK cells, and antigen-presenting cells (APCs) and affect their functioning, especially by skewing responses towards IL-17 production. It has proven difficult to confirm or refute these three hypotheses. The discovery that susceptibility to AS is influenced by polymorphisms in the ERAP1 molecule, but only in HLA-B27 ^+^ subjects, promised to shed some light on the issue ^7^. ERAP1 is a protease whose function is to trim antigenic peptides to appropriate sizes to fit into class I MHC molecules. Would the polymorphisms in ERAP1 change the repertoire of peptides presented by B27, affect assembly in the ER (and hence misfolding), or alter the expression of surface multimers? It was reported that alleles of ERAP1 that afforded protection from AS showed defective protease function. This appeared to be evidence against a role for either ER stress or the expression of multimers in SpA pathogenesis, since poorly functioning ERAP1 would be predicted to impair B27 assembly and hence increase ER stress. Likewise, poor assembly of B27 with optimal peptides might lead to increased expression of unusual forms of B27 at the cell surface. Thus, on balance, the observations on ERAP1 were taken as evidence in favour of a role for B27 presenting a pathogenic peptide, since inefficient ERAP might avoid generation and presentation of such a peptide.

There was, therefore, a good deal of interest when it was reported by Reeves *et al.* ^8^, firstly, that ERAP1 was more polymorphic than previously suspected – 13 alleles were described – and, furthermore, that when subjects were characterised according to the two alleles (co-dominantly expressed) that they inherited, it was possible to classify them accurately as AS patients or healthy controls. The AS patients had combinations of alleles that were defective in their protease activity – either inefficient trimmers of peptides or hyper-efficient trimmers. Thus, this study linked susceptibility to AS with defective ERAP1 function rather than the other way round. Whilst provocative, the findings were based on sequencing studies in a fairly small number of AS patients and controls (17 and 19, respectively).

Very recently, these findings have been strongly questioned ^9^. By examining ERAP1 haplotypes (i.e. ERAP1 alleles with combinations of polymorphic amino acids at five positions) in 213 AS family trios (71 patients and their parents), supplemented with sequencing studies in 48 additional AS patients, Roberts *et al.* found only six haplotypes, with no examples of six of the haplotypes described in the previous study or of nine of the putative polymorphisms previously postulated. Only four alleles were common. There were no examples of a particular haplotype combination previously suggested to be present in over 50% of AS patients. Extending their findings by imputing alleles from SNP data available from very large numbers of patients and controls previously examined in the Immunochip study (4,230 AS cases and 9,700 controls ^10^) confirmed the finding of four common haplotypes and noted three other rare ones (<1% population frequency), including one suggested in the previous work to be common in AS. The major difference between AS patients and controls concerned a haplotype containing K528 (instead of R528) present in 33% of AS patients versus 27% controls. The arginine is associated with defective function – i.e. the defective version is more common in controls (it is protective), though, as in other studies, it is worth remembering that a majority of AS patients (60%) still have ERAP1 molecules with the “defective” R528. This new report would appear to comprehensively contradict the previous findings, and the explanation for the differences between the two studies is not yet clear.

### The effects of decreasing ERAP1 function *in vitro*

Studies on the effects of *in vitro* silencing of ERAP1 expression, to mimic the effects of hypofunctional alleles such as R528K, have also reported disparate findings. Chen *et al.* ^11^ showed a decrease in expression of B27 free heavy chains at the cell surface in monocytes of AS patients carrying protective alleles and therefore likely a decrease in heavy chain multimers, though this was not shown directly. Importantly, silencing ERAP1 in particular B27-transfected APCs (HeLa and C1R cells) had the expected functional effects of lower levels of multimer expression. Multimer expression engages KIR (specifically KIR3DL2) on T cells and NK cells, increasing IL-17 production by T cells and survival of NK cells. When ERAP was silenced, both T cell IL-17 production and NK cell survival decreased. Perhaps unexpectedly, these results suggest that effective ERAP1 function, and hence optimal peptide loading of B27 molecules, leads to more expression of the forms of B27 that can be recognised by KIR3DL2 on T cells and NK cells. The result raises the possibility of treating AS by inhibiting ERAP1, i.e. mimicking the effects of the hypofunctional protective alleles.

In contrast, Tran *et al.* ^12^ reported that knockdown of ERAP1 expression led to greater expression of surface B27 dimers, although these experiments were carried out in U937 cells, a more typical monocyte-like APC line. Which of these contrasting findings on the effects of decreased ERAP1 expression applies physiologically is unknown. It will depend on the type and situation of the critical APC involved in AS pathogenesis. If this is more like U937, the results of Tran *et al.* would speak against a role for the expression of surface B27 multimers in pathogenesis (since protective ERAP1 alleles increase their expression). Conversely, if an APC in the gut or even enthesis has properties more similar to the cells used by Chen *et al.*, B27 multimers might still play an important role.

## The stimulus to IL-23 production in spondyloarthritis

The most common stimulus to IL-23 production is the interaction between APCs and micro-organisms, whether pathogens or normal flora. Thus, in SpA there may be an encounter with a pathogen (e.g. Salmonella), as seen in reactive arthritis, or an alteration in the relationship between normal bacterial flora and APCs in the context of gut or skin inflammation, as seen in SpA associated with inflammatory bowel disease and psoriasis, respectively. There has been a great deal of speculation about the role of the microbiome, especially the gut microbiome, in the pathogenesis of AS, given the high proportion of patients with subclinical gut inflammation.

### The microbiome in an animal model of SpA

An opportunity to monitor changes in the gut flora as SpA evolves is given by the B27 transgenic (B27tg) rat, which over-expresses human B27 heavy chain and β _2_ microglobulin and develops many features of SpA, especially inflammatory bowel disease and arthritis/enthesopathy. Asquith *et al.* ^13^ recently analysed caecal microbiota sequentially from weaning to development of colitis and arthritis. Changes between wild-type and B27tg rats’ microbiota were first evident at 10 weeks, around the time that colitis develops. There were increases in certain bacterial species ( *Proteobacteria* and *Akkermansia muciniphila*) with decreases in others ( *Firmicutes*). At early time points (3 weeks post weaning), the bacterial flora in both wild-type and B27tg littermates resembled the abnormal flora already noted in adult B27tg rats, and this reflected the fact that they were all bred from B27tg mothers and therefore initially inherited their maternal flora. However, this potentially abnormal flora did not persist in any rats at 6 weeks. Thus, some feature of the expression of B27 led to changes in the microbiota by 10 weeks, and analysis of cytokine mRNA expression in the gut revealed increases in TNFα and IL-1β, together with increases in anti-microbial peptides. This was before any colitis or changes in microbiota were observed. Hence, it appeared that over-expression of B27 led to an exaggerated gut inflammatory response, which in turn altered the microbiota and allowed colitis to develop. At these later stages, increased expression of IL-23 and IL-17 were evident, initially in the gut mucosa and later the draining lymph nodes, perhaps driven by the altered microbiota or amplified by them. *A. muciniphila* was particularly highlighted in this respect, since local expression of IL-23, IL-17, and IFNγ correlated with levels of this organism. Elevated levels of this organism were also seen in rats developing arthritis as compared to those that did not. Although increased carriage of this organism has also been reported in some SpA patients, its aetiologic role in SpA is not clear, and its presence might simply be a marker of gut inflammation.

### The microbiome in human SpA

Whilst there are relatively few available data on the microbiome in human SpA, one study in 2015 showed clear differences in the microbial population of the terminal ileum of early AS patients when compared to healthy controls ^14^. There was a higher abundance of five families of bacteria, some of which have previously been associated with colitis and Crohn’s disease (CD). In relation to CD, a very recent report compared a group of patients with CD who were divided into those with or without arthritis ^15^; those with arthritis had average BASDAI scores of >4. All patients had active bowel disease, had not received recent antibiotics, and had intact ileocaecal valves. Interestingly, patients who were HLA-B27 ^+^ were excluded from the group, apparently to decrease the proportion with axial SpA, though of course only 50% of those with CD and axial SpA will be B27 ^+^, and no radiographic data on sacroiliitis were presented. Using 16S rRNA sequencing of faecal bacteria did not reveal differences between the two groups, though CD patients differed from those with ulcerative colitis. However, when instead bacteria that had acquired a coating of IgA were investigated, there was an increase in the abundance of Enterobacteriaceae in patients with arthritis, and specifically of adherent-invasive strains of *Escherichia coli*. Whilst the result was statistically significant, the difference was largely due to much higher levels in approximately one-third of those with arthritis. It has previously been shown that IgA coating of bacteria is a good marker of those species within the gut flora that have engaged the attention of the immune system and are therefore more likely to be pathogenic. Additional evidence of the possible effects of these strains of *E. coli* came from studies in mice in which the bacteria were shown to exacerbate various IL-23-driven pathologies, including arthritis and colitis. This is a provocative and initial report, and it will be interesting to see the same approach applied to additional cohorts of SpA patients. SpA has long been associated with elevated levels of IgA, with raised IgA specific for the enteric triggers of reactive arthritis being especially notable.

## Conclusions

Together, the selection of recent papers that I have highlighted in this review shed additional light on the current central hypotheses on the pathology of SpA. They provide important additional evidence of an IL-23-responsive cell in the enthesis, clarify the role of ERAP1 polymorphisms in susceptibility to SpA, and investigate the possible implications of those polymorphisms for HLA-B27 assembly and expression. Lastly, the interplay between B27 expression and microbiota has been examined in one of the most compelling animal models of human SpA, whilst other reports have provided additional hints of the role of the microbiota in human SpA. Further developments on each of these aspects are likely to follow soon and are eagerly awaited.
